# Dental and Nondental Stem Cell Based Regeneration of the Craniofacial Region: A Tissue Based Approach

**DOI:** 10.1155/2016/8307195

**Published:** 2016-04-10

**Authors:** Declan Hughes, Bing Song

**Affiliations:** School of Dentistry, Cardiff University, Heath Park, Cardiff CF14 4XY, UK

## Abstract

Craniofacial reconstruction may be a necessary treatment for those who have been affected by trauma, disease, or pathological developmental conditions. The use of stem cell therapy and tissue engineering shows massive potential as a future treatment modality. Currently in the literature, there is a wide variety of published experimental studies utilising the different stem cell types available and the plethora of available scaffold materials. This review investigates different stem cell sources and their unique characteristics to suggest an ideal cell source for regeneration of individual craniofacial tissues. At present, understanding and clinical applications of stem cell therapy remain in their infancy with numerous challenges to overcome. In spite of this, the field displays immense capacity and will no doubt be utilised in future clinical treatments of craniofacial regeneration.

## 1. Introduction

The human body is capable of phenomenal repair; however this process is flawed; the swift production of scar and fibrotic tissue closes wounds rapidly but prevents proper recovery of function. Regenerative medicine is a rapidly expanding field concerned with “the process of creating living, functional tissues to repair or replace tissue or organ function lost due to age, disease, damage, or congenital defects” [1]. Attempted regeneration of physiological structures is concerned with the use of progenitor and stem cells, tissue engineering, and scaffolds as well as use of cellular signals [2, 3].

This paper provides a brief introduction to stem cell therapy before outlining such stem cell based regeneration of craniofacial tissues, in a tissue type based fashion. The sections are divided into mineralised tissues, dental tissues, soft tissues, sensory tissues, and exocrine glands. These sections are then further divided into subsections discussing stem cell therapy in regard to each of the individual tissue types. Following the dialogue of stem cell tissue regeneration is a brief paragraph conferring the frontier of stem cell therapy and what upcoming research may discern.

*(1) Stem Cell Source and Type*. Stem cells are a cell type capable of self-renewal and natural or induced differentiation into multiple mature cell types [4]. Tissue engineering harnesses these unique characteristics in order to regenerate functional aesthetic tissues [5]. The stem cell source and characteristics of the cell are hugely relevant to its ability to regenerate the tissue of choice. SCs are classified by their differentiation potential, their tissue, and individual of origin (Table 1). Classifying stem cells by origin involves firstly defining cells by the individual they were obtained from and then from their native tissue.

Choosing a stem cell type for regenerative purposes must involve careful consideration of source and characteristics in order to maintain the cells natural propensity and differentiation potential; however setting strict criteria is idealistic and stem cell selection must include factors such as feasibility, expansion potential, teratogenicity, and morbidity of harvest.

Adult stem cells are immunosuppressive and can be obtained with relative ease, however not without their drawbacks. Adult stem cells are difficult to expand ex vivo and have limited differentiation abilities [6, 7]. The majority of craniofacial structures derive from mesenchymal tissues. Therefore mesenchymal stem cells (MSCs) are of major interest in regenerating damaged or diseased craniofacial structures [4]. MSCs can be obtained from a wide variety of tissues such as bone marrow cultures, adipose tissue, muscle, skin, and PDL [8]. MSCs obtained from sites other than bone marrow show similar characteristics, for example, ASCs which possess relatively analogous multipotent characteristics of BM-MSCs but less morbidity from extraction and can be obtained in much larger quantities leading to less ex vivo expansion [9].

**Figure 1:**
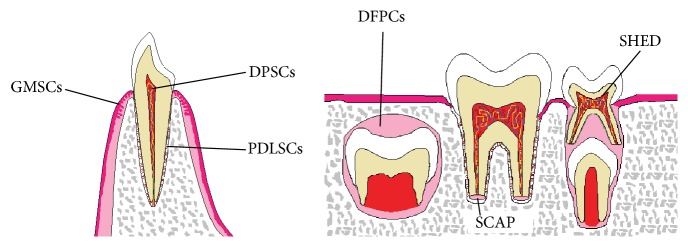
Illustrated are the separate origins of dental stem cells.

Mesenchymal stem cells of dental tissues are of neural crest cell origin and possess particular relevance to regeneration of the craniofacial region as they have a shared embryological origin [1]. Dental stem cells consist of Dental Pulp Stem Cells (DPSCs), Stem Cells from Human Exfoliated Deciduous (SHED) teeth, Stem Cells from Root Apical Papilla (SCAP), Periodontal Ligament Stem Cells (PDLSCs), Dental Follicle Precursor Cells (DFPCs), and Gingiva Derived Mesenchymal Stem Cells (GMSCs) (Figure 1) [4, 10].

Dental stem cells have displayed excellent pluripotency with the ability to differentiate into endodermal, mesodermal, and ectodermal tissue lineages providing huge regenerative scope. DPSCs may be harvested relatively noninvasively and have shown the ability to differentiate into a wide variety of tissues such as insulin producing pancreatic islet-like aggregates which may present valuable use in the treatment of diabetic children. DPSCs have also shown the ability to differentiate into hepatocyte like cells and to improve cardiac function in a murine infarct model [10]. Further proof of the value of dental stem cells in regenerative medicine was demonstrated by the differentiation of DPSCs into smooth muscle cells which holds great promise in noninvasive bladder tissue engineering but may easily be translated to other tissues such as gastrointestinal or respiratory tracts [11]. Indeed, dental stem cells possess a wide and diverse range of regenerative possibilities (Table 2).

The differentiation potential of a stem cell type dictates its applications within the regenerative field. Nonadult stem cells possess broader differentiation potential relating directly to more powerful regenerative characteristics [6]. Embryonic stem cells are totipotent meaning that they have the capacity to differentiate into embryonic and extraembryonic tissues. Despite possessing massive regenerative potency, their relevance is limited due to difficulties in accurate manipulation towards a particular cell lineage and by chromosomal instability of stem cell cultures in vitro. The extended in vitro culture time of these cell types results in a greater likelihood of structural chromosomal abnormalities leading to undesirable consequences in vivo such as teratoma or teratocarcinoma formation [6, 12]. There are also numerous ethical and regulatory issues governing the use of embryonic stem cells [6]. Foetal and perinatal stem cells display limited expansion and decreased potency compared to embryonic stem cells; however harvesting such cell types is somewhat less controversial [6].

To overcome some of the barriers of adult and nonadult stem cell sources, induced pluripotent stem cells were developed. These are terminally differentiated somatic cells which are generated via genetic reprogramming to revert back to a plastic multipotent cell type. These stem cells possess unlimited expansion opportunities in vitro and may differentiate into cells of any tissue type [6]. While studies have shown iPSCs to be hugely promising, more research is needed to optimise their reprogramming to prevent an immune response. Indeed while all stem cell types have limitations, there is much on-going research on controlling pathways and cell differentiation behaviour at a molecular level to improve characteristics which will aid their regenerative characteristics and reduce their tumourigenicity [6, 12].

While the harvesting and manipulation of stem cells have shown intimidating promise in the field of regenerative medicine, there may arguably be a more efficacious method of manipulating the stem cells already present in the host. Intrinsically controlling stem cells through the use of local growth factor delivery and planned control of signalling pathways allows for increased native stem cell migration and improved healing times without the need for invasive harvesting procedures. The use of growth factors and other small molecules can optimise the healing process through early activation of repair mechanisms and suppression of harmful inflammatory or immune responses [13, 14]. Optimising native stem cell populations occurs through intelligent growth factor manipulation directing cell homing and guiding the stem cell niche [13, 14]. Cell homing consists of supplying factors at the correct time to support proliferation, differentiation, and migration of SCs with the aim of improving endogenous stem cell movement to the injury site. Another technique is the targeting of the stem cell's microenvironment with direct delivery of growth factors resulting in increased SC motility, division, and maturation rates [13, 14]. While these methods may present less treatment morbidity, they may be limited by decreased native stem cell populations, therefore supporting the idea of a combination therapy approach in order to obtain the most effective treatment results.

*(2) Harnessing Scaffolds and Biomaterials to Improve the Regenerative Capacity of Stem Cells.* The actions of cells are entirely dependent on their surrounding cells and their extracellular matrix. Cell-ECM interactions govern migration, proliferation, adhesion, and even gene regulation. The use of scaffolds and biomaterials in tissue engineering and regenerative medicine allows us to influence cells in such a way as to increase their regenerative capabilities. Through biomaterial manipulation to effectively mimic the specific ECM properties, it is possible to synergistically drive stem cell fate towards a desired lineage. Intelligent biomaterial design should aim to closely mimic the microenvironment of its desired physiological niche in order for stem cells to deduce the instructive effect of the biomaterial and hence follow its desired cell lineage. Stem cells will detect the specific material properties of a biomaterial and code for specific biochemical signals affecting its regenerative properties and ultimate fate [15]. Therefore scaffold properties such as topography, chemistry, porosity, material choice, and biocompatibility hugely affect biomaterials regenerative capabilities.

Currently it is widely accepted that the cell-substrate interface is the gateway through which cell lineage is decided upon. Biomaterials harness this gateway through altering topography and chemical structure to direct stem cell adhesion, migration, differentiation, and proliferation. Material surface topography may be altered via various methods to provide the micro- or nanotopographical surface roughness and distribution desired to direct cell lineage. Studies have shown stem cells to align and orientate themselves depending on topography [16]. Surface topography may also be altered through various coatings such as laminin or polylysine mimicking the architecture of the basement membrane to aid in terminal differentiation of stem cells. Surface coatings also allow for a combined approach utilising materials of suitable mechanical properties to be layered with a biomaterial of suitable chemical properties. The surface area of the cell-substrate interface is directly affected by the relative porosity of a material. A highly porous communicating geometry will allow for cellular penetration into the scaffold, uniform distribution, and angiogenesis [15, 16]. Pore manipulation is hugely important in scaffold design as it allows communication to the native tissues. Effective pore design should allow for cellular migration, neovascularisation, and the diffusion of substrates and nutrients to fuel stem cell activity.

The biocompatibility of a scaffold material is essential to its function and successful application [17]. It is critical that scaffold implantation causes minimal biological response which may upset the artificial stem cell niche and hence hinder the regenerative process. Biocompatible scaffolds must also effectively mimic host tissues in order to allow native cellular activity and signalling without interruption. For obvious reasons long-term presence of a scaffold is hardly ideal; therefore degradability of biomaterials is a highly attractive characteristic. Scaffold degradation must be a controlled process, occurring at a defined known pace matching tissue growth rates, while releasing nontoxic products that are safely metabolised without causing any form of inflammatory response [15, 18]. As the degradation rate of many polymeric scaffold materials is well understood, it is possible to entrap molecules into the biomaterial so that they may be passively released at the required site at a defined rate. This property has huge applications for stem cell therapy as it allows for modulated drug delivery systems and the incorporation of specific growth factors to target specific stem cell lineages [19]. This intelligent incorporation of bioactive molecules into scaffolds presents many advantages such as increasing control over regeneration as well as increasing the functionality and strength of the final tissue [20]. Growth factors can be sequestered into scaffolds by two main techniques, chemical attachment or physical encapsulation [20]. Each of these two techniques allows for a localised interaction between the desired cells and the entrapped growth factors to influence differentiation for the targeted expansion of a specific stem cell lineage [20].

There is an ever increasing array and variety of scaffolds being invented and innovated upon. The majority of scaffolds used in tissue engineering and regenerative medicine tend to be polymer based as they can form strong rigid structures but eventually degrade into monomers which will be metabolised or excreted. Polymers can also be modified by the addition of substances related to the particular tissue being regenerated. An example of this is the addition of hydroxyapatite and tricalcium phosphate to form a tough composite/polymer hybrid scaffold intended for bone regeneration [18]. This substance has improved biocompatibility and buffers the acidic pH during resorption which would otherwise inhibit the healing process [18]. However this is just an example and there is a wide array of scaffold materials and fabrication techniques available and being experimented upon, each possessing its own unique characteristics and advantages. Therefore evaluation of scaffold type for tissue regeneration may best be performed depending on the discrete clinical requirements.

## 2. Stem Cell Based Regeneration of Mineralised Craniofacial Tissue

### 2.1. Stem Cell Based Skeletal Tissue Engineering and Activation of Endogenous Healing through PEMF

Trauma, ablative surgeries, or congenital deformations can result in reduced or missing bone. Regeneration of this bone is essential for proper soft tissue regeneration, muscle function, and restoration of the facial appearance. Severe damage to the craniofacial bones not only causes physiological pain but also may lead to emotional and psychological damage. The need for craniofacial bone regeneration not only is an ethical requirement but also is emerging as a hugely remunerative market with around $390 million US dollars spent on craniofacial bone trauma in the US alone in the year 2010 [21].

Current treatments of craniofacial bone defects involve bone grafts and “distraction” techniques. There are several types of bone grafts available: autogenous, allogenic, and alloplastic [21]. Autogenous and allogenic bone grafts are associated with superior osteoconductive properties but are associated with morbidity of donor site and increased infection rates. There is much research currently on alloplastic grafts/scaffolds which consist of forming a synthetic bone-like matrix which can be produced with the necessary structural, chemical, and physical qualities cost-effectively and in abundance. However current materials currently lack the high levels of integration seen in biological grafts [21].

Distraction osteogenesis (DO) is implicated in the treatment of many congenital craniofacial syndromes resulting in hard tissue defects such as “maxillofacial microsomia, micrognathia, and temporomandibular joint ankylosis” and “midface hypoplasia, maxillary deficiency, zygomatic deficiency craniosynostosis, cleft lip and palate (CLP), and transverse discrepancies” [22]. The technique has been shown to restore aesthetics in defects; however complications occur in “upwards of 35%,” formed bone is 40% weaker, and there is a high morbidity associated with the procedure [1, 4, 23]. The limited outcome obtained by such a procedure highlights the current potential for regenerative techniques to improve procedures by lowering patient morbidity and raising the overall efficacy of the treatment.

Combination techniques utilising stem cells and proven classical techniques possess potential in bone regeneration following radiotherapy which can induce degradation of bone and hinders wound healing [24]. A study carried out by Deshpande et al. shows that the BMSC/XRT/DO-treated animals demonstrated a statistically significant increase in the Y, UL, and FL in comparison to the XRT/DO group of 1,009%, 1,679%, and 5,396%, respectively. While this is a statistically significant improvement it must be noted that it was noticeably less than the control nonirradiated mandibles which underwent DO without the use of BMSCs [24].

While using stem cells with current techniques has shown promise, there is still a high morbidity associated with procedures such as distraction osteogenesis. Therefore the use of scaffolds seeded with growth factors and SCs to rapidly repair skeletal defects is highly anticipated [25]. Scaffolds come in a variety of forms and act as osteoinductive tissue engineered extracellular matrix which supports the initial growth and development of regenerating bone during osteogenesis [25].

Such an example of using stem cell therapy to ameliorate skeletal defects was carried out by Farré-Guasch et al. They suggested using a 1-step surgical maxillary sinus floor elevation to allow for dental implants in the treatment of maxillary atrophy. This treatment aims to decrease expense and the need for additional surgical intervention which would increase morbidity. This technique involves liposuction to obtain a stromal vascular fraction of adipose stem cells (ASCs) and relevant growth factors (BMP-2) which are then seeded onto a calcium phosphate ceramic scaffold which is then injected into the osseous defect. This technique could provide a viable, more efficient method of treating the increasing prevalence of maxillary atrophy [26].

Stem cell usage has enormous potential to improve current treatment of congenital craniofacial defects such cleft lips and palates, craniosynostosis, Treacher Collins syndrome, and Pierre-Robin syndrome. Cleft lips/CLP represent ~50% of all craniofacial congenital defects, each case requiring complicated treatments from a multidisciplinary team. Individuals afflicted by clefts exhibit decreased alveolar bone growth and decreased sagittal maxillary growth [27]. Common treatment of this defect involves maxillary advancement and alveolar bone grafting; however these treatments while effective have their disadvantages. Additionally the use of scaffolds or alloplastic grafts is normally unsuitable for treatments in growing children as fixed appliances do not evolve with the surrounding growing tissues [27].

Reconstructing craniomaxillofacial hard tissue defects is an extremely challenging objective; therefore inclusion of stem cell therapy to aid treatment outcome is entirely necessary. Using ASCs and in some cases supplemental BMP-2, Sándor et al. sought to reconstruct cases of severe frontal sinus infection in need of obliteration, cranial defects in need of a cranioplasty, and mandibular defects resulting from recurrent ameloblastomas in need of resection and chronic nasal septum perforations [28]. Many of the cases have had previous conventional treatments which were regarded as unsuccessful. The patients who underwent frontal sinus treatment displayed no recurrent infection and “remarkable” bone formation and remained asymptomatic. Treatment of the cranial and mandibular defect cases was successful without presenting any complications. Two of the 3 mandibular defect patients then opted for implants which were successfully osseointegrated and managed in masticatory loading [28]. This study relatively uniquely also examined the cell markers of their adipose aspirated to ensure it was of mesenchymal origin and not of haematopoietic and angiogenic origin. This ensured a high commitment to forming tissues of a mesenchymal origin (i.e., bone) and may have been a controlling factor in these studies [28].

While the following techniques have all shown positive impacts on healing there is still morbidity associated with undertaking an invasive surgical approach to encourage healing. An interesting technique which is becoming increasingly accepted in mainstream medicine is the use of low-frequency pulsed electromagnetic fields (PEMFs). This treatment option is attractive as it is noninvasive and painless; PEMFs work through creating an electrical field of 1–100 mV/cm to induce bone and vascular growth through increased native growth factor expression [29]. Controversial variable results have been obtained regarding the use of PEMFs on bone regeneration; however recent results are beginning to show that PEMF treatment is becoming a viable, effective, and feasible treatment option [30].

The feasibility of this treatment combined with the elimination of infection risk certifies PEMF as an extremely attractive craniofacial treatment option to reduce healing times, support the reconstruction of critical sized osseous defects, and so forth [31]. Experimentation was carried out using a high frequency PEMF to stimulate the differentiation of immortal osteoprogenitor cells from the calvaria of CD1 mice in vitro. While the underlying mechanisms behind PEMF enhanced bone regeneration are not entirely clear, this study has been able to show a correlation between the use of high frequency PEMF and a time related increase in various different osteogenic promoters. The use of immortal mice calvarial cells illustrates how PEMF may become a potential treatment option for craniofacial bone regeneration as embryological origin did not appear to be a changing factor [31]. However more research is required on in vivo animal models for more conclusive results to be drawn.

Current treatment of mandibular factures involves surgical intervention or interdental wiring. Both practices are associated with high morbidity; therefore the use of PEMF is ideal to reduce fracture healing time and the associated morbidity. Abdelrahim et al. investigated the clinical effects of PEMF on mandible fracture healing. Fractures were reduced and maxillomandibular fixation was carried out using arch bars and surgical wiring. PEMF was found to significantly increase bone density, reduce resorption, and decrease pain intensity [32]. This study highlights how PEMF could be a very viable treatment option for improving healing rates in craniofacial fractures. However this study falls short on a number of levels such as a small patient group, patients suffering from postoperative infection, and patients undergoing incomplete treatment. Larger treatment groups and more randomised control trials are needed to truly analyse its effectiveness and feasibility [32].

The installation of dental implants can be a complicated morbid procedure especially in cases complicated by disease or low bone levels; therefore there is much interest in methods of improving the osseointegration of implants. Due to PEMFs promotive effect on osteogenesis and osteogenic differentiation and the efficacy of its use on the mandible, Wang et al. set out to determine whether PEMF could result in the increased osseointegration of different types of titanium dental implants. The experimental PEMF group displayed improvements in the following areas: protein adsorption, cell adhesion, cell proliferation, cell morphology, ALP activity, and ECM mineralisation. Analysis of gene expression shows that PEMF has a stimulatory effect on the expression of all osteogenesis related genes [33]. This experiment shows PEMF to be a powerful tool, capable of greatly enhancing biocompatibility and osseointegration in vitro through cellular manipulation and gene regulation. Wang et al. discuss the fact that this increase in compatibility could be due to the PEMF changing the electrical potential of the implant surface which could possibly affect polarisation of the cell membrane enhancing adsorption [33]. The results of this study suggest that PEMF could be a potential accomplice to dental implant treatment where poor integration may be of concern; however further in vivo experimentation is needed to evaluate efficacy.

The discussed studies using MSCs and PEMF have shown encouraging results. Experimental studies are now also beginning to investigate the use of dental stem cells in repairing osseous defects with extremely promising initial results. Moshaverinia et al. reported that SHED and DPSCs had equal results to MSCs when attempting to regenerate a significant mandibular osseous defect in a canine model. Also suggested were comparable immunosuppressive activity of the dental stem cells and the fact that paediatric SHED from offspring could be used in treatment of parental defects [34]. Other dental stem cell sources such as PDLSCs and GMSCs present with excellent accessibility but have been shown to possess poor osteogenic capacity compared to BM-MSCs [35]. Consequently future studies aiming to use dental stem cells to accelerate craniofacial skeletal regeneration should concentrate on the use of DPSCs due to easy accessibility and strong osteogenic properties [36, 37]. However research is required to determine whether DPSCs possess enough osteogenic potential to warrant their use over BM-MSCs.

### 2.2. Stem Cell Based Joint Regeneration, a Possibility Worth Investigating?

The reconstruction of craniofacial mineralised tissues is not just limited to bone but may also have applications in osteochondral defects. One such area in need of regenerative solutions is the TMJ. TMJ disorders cover a wide range of conditions occurring with a high incidence rate with 75% of the population showing one or more signs of joint dysfunction and 33% of the population possessing at least one symptom such as joint pain [38]. Native healing of the TMJ is reduced due to poor vascularisation linked to diminished healing, hypocellularity, and decreased progenitor cell populations. The high prevalence and associated high morbidity of TMJ disorders strike the need for regenerative treatment options [38, 39]. However treatment of TMJ disorders is complicated by several factors such as lack of explicit causation, the close integration of tissues, prevention of adhesion of structures, and finally the ability for a regenerated structure to sustain substantial masticatory and kinetic stresses [4, 38, 39].

Similarly to bone regeneration, most experimental studies have concentrated on the use of mesenchymal stem cells to regenerate the osseous structures of the TMJ. Re'Em et al. developed a MSC seeded bilayer hydrogel system containing osteoinductive BMP-4 and chondroinductive TGF-*β*1 in respective layers to implant in New Zealand white rabbits [40]. MSCs displayed the ability to differentiate into osteoblasts and chondrocytes in their respective hydrogel layers which then developed into separate distinguishable tissues. The creation of a multilayered TMJ-like construct is a step towards functional TMJ regeneration; however, much more research is required before mechanically satisfactory constructs could be successfully placed in vivo [40, 41].

Using a similar model to Re'Em et al.'s, an in vivo model of a dental stem cell seeded hydrogel system was successfully carried out using GMSCs and PDLSCs in an in vivo mouse model to stimulate ectopic chondrogenic regeneration. The GMSCs and PDLSCs were encapsulated in a TGF-*β*1 ligand before being seeded into the RGD coupled alginate which was then implanted into the dorsal surface of beige nude mice [42]. Analysis revealed differentiation into cells morphologically similar to chondrocytes and the endogenous release of chondrogenic growth factors, BMP-4 and FGF-2 resulting in the deposition of cartilage-like tissues containing proteoglycan and collagen type II. PDLSCs were noted as having even higher production of collagen type II than BM-MSCs, therefore providing support to the notion that stem cells of a neural crest origin may be preferential candidates for TMJ regeneration [42].

However regeneration of the TMJ is not solely concerned with the mineralised structures of the joint. The TMJ is composed of multiple tissue types all interacting to allow movement of the bicondylar hinge. One of these components is the meniscus or disc which acts to reduce friction and provide cushioning between the mandible and the articular fossa. In TMJ defects this disc is commonly displaced and/or damaged. Current treatments of TMJ discs involve removal and placement of artificial prostheses; however a review carried out by Hagandora and Almarza dismisses the placement of artificial TMJ claiming few long-term benefits, the possibility for further degeneration, and failure to treat joint dysfunction. Tissue engineering techniques have the potential to become a more suitable solution for repair of TMJ defect and regeneration of the damaged TMJ [43]. One of the initial regenerative attempts using stem cells was carried out using TMJ derived synovial stem cells seeded onto a fibrin/chitosan scaffold hybrid implanted into nude mice. The experimental construct displayed improved cell adhesion and proliferation as well as increased expression of GAGs and collagen type 1 [44]. This pilot study suggests a potential treatment model for TMJ disc perforation; however more animal models are needed to discover the most effective scaffolds and stem cell choices.

Future studies should concentrate on exploiting a stem cell type with both chondrogenic and osteogenic properties which can be held in an engineered construct containing molecules to direct stem cell fate towards the desired linage in a spatial arrangement such as the study carried out by Re'Em et al. Dental stem cells have been shown to possess both chondrogenic and osteogenic properties and future research may reveal them as an attractive stem cell source for TMJ reconstruction [11, 42]. Certain challenges must be overcome before such therapies have any clinical applications such as the fact that guaranteeing constructs can sustain the substantial mechanical and kinetic forces generated during functioning and the ability to facilitate natural remodelling of the joint postoperatively [4].

## 3. Potential Applications of Stem Cell Therapy in Dentistry

### 3.1. Tooth Regeneration, the Ultimate Goal of Dental Science

Teeth play a pivotal role in the oral cavity affecting all major oral functions. Any loss of teeth can affect an individual's diet, social habits, psychological health, communication skills, and so forth. Cases where violent trauma or oral neoplasm excision has occurred are in great need of a suitable tooth regenerative strategy. Also in severe need of a regenerative strategy are patients suffering from congenital defects such as amelogenesis, ectodermal dysplasia, and Rieger syndrome [45]. Tooth regeneration presents similar challenges to that of bone regeneration such as the need to sustain considerable forces. However, to properly recreate a functional tooth, there is the additional challenge of regenerating multiple hard and soft tissues in harmony. While challenging, regeneration of dental structures is a necessary progression from current treatments which are imperfect and unequal to that of the natural tooth. Common restorative procedures have high failure rates which result in a continual cycle of further tooth destruction. These treatments may also have personal requirements which are commonly unavailable such as unsuitable bone levels for dental implants.

An interesting yet ambitious method of tooth regeneration was developed by Sonoyama et al. In their study they built up a bioroot formed of apical papilla stem cells seeded into the HA-TCP complex coated in a “Gelfoam” containing PDLSCs which was then implanted into a lower incisor socket of a mini pig [46]. After 8 weeks the bioroot had formed cementum and Sharpey's fibres attachment; finally after 3 months, a porcelain crown was fitted to the bioroot [46]. While the crown showed sufficient strength to support crown and carry out normal masticatory function, it still did not possess the loading strength of a comparable natural tooth and their method of cell delivery has low clinical potential due to costs [46, 47]. However the employment of multiple stem cell types to regenerate their propensive structures may be necessary in future studies to acquire a model with clinical suitability.

Kim et al. attempted a more feasible method using cell homing to regenerate human mandibular molars in Sprague-Dawley rats. This was carried out by implanting a 3D printed PCL-HA scaffold with microchannels seeded with BMP-7 and SDF-1 into the dorsum of the rats. BMP-7 was chosen for its ability to encourage mineralisation, while SDF-1 was selected for its ability to bind to the chemokine receptor CXCR-4 which is present on endothelial cells and bone marrow SCs/progenitors [47, 48]. This study significantly generated alveolar bone, pulp, cementum, PDL, and dentine, resembling tissues which were richly vascularised, and it proved that cell homing could be a viable future method of tooth regeneration [47, 48].

Despite Sonoyama et al. and Kim et al. showing successful tissue regeneration, they failed to mimic the complex natural tooth tissue composition and structure. Oshima et al. sought to overcome this by bioengineering a molar tooth germ. The construct would consist of epithelial and mesenchymal components placed in a plastic size control device to implant in the subrenal capsule of a mouse where it would progress and develop [49]. Once established the bioengineered tooth unit was implanted into an extensive alveolar defect generated in the lower molar region of a mouse where it possessed proper occlusion. Surface hardness of the bioengineered tooth tissues was comparable to that of a normal mouse molar [49]. Experimental orthodontic procedures demonstrated alveolar remodelling with the presence of osteoblasts and osteoclasts on the respective tensive and compressive sides indicating proper PDL integration. Successful integration of the bioengineered tooth was shown through the development of nerves and blood vessels to the pulpal region and some regeneration of the alveolar bone defect [49].

Oshima et al.'s work illustrates a potential viable model for whole tooth regeneration. The integration and effectiveness of this model could potentially be further increased by developing a method of tooth germ development in a supersaturated environment and the incorporation of growth factors to improve alveolar bone regeneration. It is essential that alveolar bone regeneration remains closely associated with tooth regeneration as the future clinical uses of tooth regeneration will remain adjunctive to treatment of periodontal disease.

Honda et al. also attempted to overcome the challenges of whole tooth regeneration using a unique scaffold construct and cell seeding. Postnatal epithelial enamel organ cells and mesenchymal dental papilla cells were obtained from a porcine developing 3rd molar tooth in early crown formation [50]. The experimental construct was formed by seeding the mesenchymal cells onto a collagen sponge scaffold and then later seeding the high density epithelial cells atop the mesenchymal cells to allow for direct cell-cell interactions [50]. After implantation into immunocompromised rats, tooth germ structures were apparent in all implants with odontoblast and ameloblast-like cells secreting enamel- and dentine-like structures. The concept of this study was aiming to illustrate that, with sufficient cell-cell communication, tooth shape and tooth number can be controlled without the need for complex signalling factors or intervention. These cell-cell interactions are thought to be responsible for the increased rate at which the regenerated tooth developed [50].

This study illustrates the need to further investigate macro- and micromolecular signalling mechanisms between these tissues in order to maximise both the efficiency and structure of the final product. Furthermore future studies should also aim to develop intelligent scaffolds incorporating growth factors to encourage cell lineage towards its desired fate while controlling for the appropriate amount of mineralisation in each tissue type. Growth factor incorporation seems to be a necessity for whole tooth regeneration due the varied tissues and complex nature of signalling involved.

Regeneration of the whole tooth is important where teeth are missing or damaged beyond repair. However in many cases only parts of the tooth are damaged such as in trauma, caries, and pulpitis, and efforts must be made to develop regenerative strategies for treatment applicable to these defects. The tooth and in particular the dentine-pulp complex possess natural regenerative reactions when placed under threat such as the recruitment of progenitor cells, that is, the formation of reparative dentine and remineralisation. Even current techniques such as the use of calcium hydroxide in pulp-capping are related to reparative dentinogenesis. While the tooth possesses an intrinsic protective ability to defend itself against attack, it is often insufficient. Therefore with further elucidation of the chemotactic driving signals, it may be possible to therapeutically exploit and manipulate this mechanism to provide an efficient clinical treatment option [51]. Another possibility which was discussed was the harvesting of adult dental stem cells from locations elsewhere in the body and having them differentiate into odontoblast-like cells to direct dentine bridge formation [52].

While the dental pulp complex has some innate regenerative capabilities, enamel does not due to the loss of its formative cell, the ameloblast, upon eruption. Current restorative treatments involve the use of engineered materials. These materials possess similar but inferior mechanical and physical characteristics resulting in inadequate longevity. Regenerative techniques could potentially one day provide us with “biobonded” enamel/dentine inlays and eliminate the need for secondary restorations. New clinical techniques could culture epithelial cell rests of Malassez in the aim of differentiating them into ameloblasts, which could produce sound enamel when deposited onto an appropriate scaffold [52].

Using cells from a newborn mouse enamel organ and ameloblast-like cells, Huang et al. sought to use stem cells to develop enamel-like structure. These cells were cultured onto “a branched peptide amphiphile containing the RGD peptide epitope” to facilitate stronger adhesion and due to its ability to self-assembly into nanofibres [52, 53]. The peptide amphiphile and cell cultures were then aggregated and implanted into the enamel organ of a mouse incisor [53]. Analysis of the in vivo and in vitro experiments carried out revealed expression of enamel matrix proteins such as amelogenin, differentiation of enamel organ cells into ameloblasts, and increased mineral deposition (only confirmed in vitro) [52, 53]. This study provides further support to the use of dental stem cells for enamel regeneration. However despite laboratory successes, there is still huge amounts of research needed before regenerated enamel could ever possess feasible clinical potential.

### 3.2. Stem Cell Based Regeneration of Oral Mucosa

The oral mucosa and periodontal tissues have extraordinary abilities of self-regeneration, much higher than that of comparable tissue such as that of the skin. This is thought to be due to the unique populations of progenitor and stem cells that exist within its make-up [54]. Oral mucosa possesses extremely good healing capabilities with reduced scar formation and accelerated healing time due to decreased levels of inflammatory cells and cytokines, as well as a decrease in the ratio of TGF*β*-1 : TGF*β*-3. While oral mucosa possesses good regenerative characteristics, there is need for new regenerative techniques. Current treatments such as skin grafting have noted morbidities including oral hair growth and excessive keratinisation, and with increasing rates of oral cancer and a growing population with increased periodontal disease susceptibility, there is a definite need for regenerative treatments concerning oral tissues [55].

One of the primary concerns in regenerative medicine when attempting to regenerate functional tissue is where and how to obtain a suitable stem cell population. In the case of oral mucosa, Davies et al. found there to be an easily isolatable population of progenitor cells of neural crest cell origin within the lamina propria of the oral mucosa [54]. The isolation of such a population holds huge potential for the future of oral mucosa regeneration and indeed regeneration within the whole craniofacial region. The isolate population was observed to be multipotent and capable of generating both mesenchymal and ectodermal cell lineages. Further research could lead to the generation of a new common source of progenitor cell isolation due to the cell's high proliferative rate in vitro, the lower amount of regulatory issues concerning adult sources of stem cells, and the low morbidity of obtaining said cells [54]. Miyoshi et al. sought to take a different approach to developing an oral mucosal stem cell population. In their study they presented the case of using oral fibroblasts over dermal fibroblasts as an alternative cell population for producing iPSCs. Their research proved that oral fibroblasts could be easily translated into iPSCs with less harvesting morbidity and improved harvest location healing [56]. Garzón et al. have suggested a potential method of oral mucosal regeneration using WJ-MSCs seeded atop a stroma containing fibrin and oral mucosa fibroblasts to induce epithelial differentiation. This construct was then grafted onto a 2.5 cm^2^ skin excision on the dorsal surface of immunodeficient athymic mice. Analysis revealed the presence of epithelial keratinocytes and well-structured defined epithelial layers consisting of basal, spinosum, granulosum, and corneum cell layers. Indeed this displays a credible treatment option for clinical use in patients with the need for large areas of oral mucosal regeneration [57].

### 3.3. Stem Cell Applications in Treatment of Periodontal Conditions

Periodontal disease is a chronic inflammatory condition of the periodontium leading to periodontal tissue destruction and a loss of attachment between the tissues and the tooth. Loss of alveolar bone, cementum, gingiva, and the periodontal ligament result in gingival recession, increased dental sensitivity, and if left uncontrolled, eventual premature tooth loss. According to the adult dental health survey of 2009, 66% of the UK population over the age of 55 display some loss of periodontal attachment. Recent studies have suggested that chronic inflammation of the periodontal tissues is associated with a number of systemic diseases such as diabetes, cardiovascular disease, respiratory disease, and preterm low birth weight [58–61]. Current strategies mainly employ preventative practices to cease disease progression and the treatment approaches used clinically have variable results with minor clinical improvements. Hynes et al. carried out a thorough review of studies using stem cells in periodontal regeneration covering BM-MSCs, DPSCs, PDLSCs, SCAP, periapical follicular stem cells, and stem cells from deciduous teeth. While several stem cell types displayed some regenerative capacity, unsurprisingly PDLSCs displayed the greatest capacity for periodontal tissue regeneration with multiple studies reporting regeneration of alveolar bone, cementum, and Sharpey's fibres of the periodontal ligament [62–64]. Hynes et al. discuss the numerous clinical issues needed to overcome before clinical translation can arise such as improved understanding of cell processes and the need for “large-scale preparation facilities.” While these issues still persist, Hynes et al. discuss a method for overcoming the issues involved with accessibility and proliferation in culture via differentiating iPSCs into MSC-like cells. MSC-like cells were able to demonstrate significantly increased mineralised tissue regeneration and the formation of PDL-like tissue in a rat periodontal defect model; however the study did not mention whether the cells contributed to the regeneration of cementum [65].

While using the fenestration type model in laboratory rats is useful in evaluating and comparing the most effective possible treatment type, there is a need for more safe clinical experimentation in humans with periodontal disease such as that carried out by Feng et al. Autologous periodontal cell progenitors (PDLPs) obtained from extracted third molars were seeded into hydroxyapatite and were implanted into 16 deep intrabony defects of pocket depth ≥6 mm in 3 male patients. Each patient showed considerably significant clinical benefit from the procedure with no inflammation in the treatment area. Analysis of PDLPs compared to PDLSCs revealed many of the same stem cell properties; however PDLPs have slightly reduced osteogenic differentiation capabilities. There is a clear need for a well-performed clinical trial with greater patient numbers utilising PDLSCs to truly determine the potential viability of this treatment method [66].

## 4. Soft Tissue Regeneration

### 4.1. Stem Cell Use in Cosmetic Dermal Regeneration

Skin plays a crucial role in regulating body temperature, protection against infection, synthesising vitamin D, sensation and appearance, and so forth. Damage to skin of the craniofacial region can occur through a number of means, for example, burns, extensive trauma, and tumours. Defects of the facial appearance can be damaging psychologically as well as physiologically as it is our most identifiable feature which is integral to effective communication. While the skin has rapid repair mechanisms, the healed product, a fibrous CT scar loses function, aesthetics, and any lost appendages do not regenerate [67]. The best most currently used method of treatment is autologous skin grafts; however the procedure experiences setbacks such as availability and morbidity of donor site and scar contracture [1]. Larger procedures suffer from more severe flaws such as the need for multiple surgeries and grafts resulting in noticeable deformities [1].

There is much research on using biocompatible synthetic and natural polymers in the replacement of autologous skin grafts [68, 69]. These new forms of dressings have enormous possibilities in regeneration as they possess the ability to be seeded with stem cells, growth factors, and skin cells such as keratinocytes and fibroblasts [68]. Studies carried out with the use of stem cells within a dermal substitute have claimed improvements in angiogenesis, collagen synthesis, and healing time while also showing a decrease in fibrosis [69]. However there are not yet any studies carried out to produce a dermal substitute capable of replacing skin grafting technology [1].

Current skin regenerative technique aims to reduce scar formation; however with the use of stem cell and regenerative technology it may one day be possible to simply avoid scar formation. Jackson et al. discuss the use of MSCs in inhibiting scar formation and their many interactions/properties which positively influence cutaneous regeneration. Despite the obvious characteristics such as direct differentiation into dermal cell types, MSCs have the ability to immunomodulate T-cell and macrophage activity, reducing inflammation and promoting fibroregulation. MSCs are also understood to produce antifibrotic factors while enhancing dermal fibroblast function promoting the formation of natural extracellular matrix and a tissue similar to its surroundings [70].

Sabapathy et al. aimed to demonstrate the ability of mesenchymal stem cells to attenuate cutaneous scarring via the use of extraembryonic MSCs obtained from umbilical Wharton's jelly (WJ-MSCs). The stem cells were then seeded onto a decellularised portion of amniotic membrane which was sutured onto the surface of a 1 cm^2^ full skin excision wound in black SCID mice. WJ-MSCs were found to have significantly increased immunomodulating capabilities in the presence of proinflammatory compounds compared to typical BM-MSCs. It is thought that the increased immunomodulatory mechanisms of the WJ-MSCs are used to augment “Scar-Free Wound Healing with Hair Growth.” WJ-MSCs and the decellularised amniotic membrane were also found to have improved mechanical properties [71]. This method of preventing scar formation displays impressive results without any morbid cell harvesting methods.

Recently discovered are leucine-rich repeat-containing G-protein coupled receptors (LGR) which are present as transmembrane markers found in stem cells throughout all epithelial tissues. The recent discovery of this marker allows for cell isolation and the utilisation of such cells in the regeneration of epithelial tissue with insufficient stem cell population [72]. Lough et al. aimed to demonstrate the regenerative capabilities of these cells through delivering LGR-6+ cells underneath 3 mm^2^ burn wounds in a mouse model. Analysis of LRG stem cells effects on gene expression reveals an increase in the release of factors such as VEGF, EGF, and other factors related to tissue regeneration [72]. The release of these factors increase angiogenesis, thereby increasing wound healing time. Analysis reveals that despite trauma and the process of wound healing the LGR-6+ cells were still viable therefore lending the idea that the cells have potential healing much larger defects than the 3 mm^2^ defects created in this study; however future research will need to confirm this. Uniquely attributed to LGR stem cells is their ability to regenerate hair follicles. This characteristic is highly valuable in regeneration of craniofacial skin due to the desire to regenerate the aesthetics of the face including facial hair [72].

The market for the rejuvenation of skin is a huge one and currently there are many products on the market with huge amounts being spent on research. Regenerative medicine is mostly concerned with regeneration of the function and aesthetics of tissues following disease and trauma; however with aging, many tissues have a marked decrease in function and in the case of skin and lose their aesthetic properties. Therefore we cannot discredit the possibilities of regenerative medicine in rejuvenation of skin and all its other less clinical and more commercial possibilities. A study by Harn et al. tested the use of peripheral blood stem cells (PBSCs) treated with granulocyte stimulating colony factor (GCSF) to rejuvenate the skin of Lanyu pigs [73]. Analysis revealed that the PBSCs migrated to dermal tissues and increased synthesis of hyaluronic acid, collagen *β*1-integrins, and elastin. This increase in synthesis of these dermal tissue components has corresponding effects on its structure such as decreased skin thickness, reduced wrinkles, and increased suppleness [73]. The results of this study are promising but are limited by the small sample size and the short analysis time which did not allow for any long-term analysis.

To define an ideal stem cell population for dermal regeneration is complicated by the nature and the severity of the injury requiring treatment. Autologous adult stem cells may not possess the expansion capabilities to provide an adequate quantity of cells whereas culturing extraembryonic stem cells may not be feasible for smaller injuries. Therefore allogeneic extraembryonic stem cell therapy may present an ideal stem cell source for widespread burn injuries due to increased immunomodulatory capacity and increased proliferation properties [74, 75], while smaller injuries may benefit from therapy using autologous ASCs which would not have to rely on tissue banking but would also benefit from immunomodulatory properties.

### 4.2. Regeneration of Fat to Reshape the Face

While replacing skin and ensuring minimal fibrosis are essential for proper facial appearance and form, fat plays a substantial role in aesthetics providing contour and volume to the face. Defects in facial fat and contour can be due to a wide variety of causes such as trauma, disease, or surgery. Currently, treatment of defects such as lipodystrophy involves fat grafting which has proven to be a very effective, long-term treatment. While this tissue is easily harvested and abundant it suffers from relatively high rates of resorption after surgery depending on technique [1]. Zhu et al. were able to show that the addition of adipose derived regenerative cells can double graft retention and improve quality and angiogenesis [76].

Similar results were found in a study by Koh et al. In this study 5 patients received ASCs and microfat grafts while the other 5 patients (the control group) received only microfat grafts. All patients in the study suffered from Parry-Romberg disease which causes progressive hemifacial atrophy. Postoperatively results were measured using a 3D camera and 3D CT scans. Treatment for all groups was successful with the ASC groups demonstrating significantly less resorption after graft. This difference is statistically significant and results in a much better graft retention resulting in better aesthetics and a more efficacious treatment [77].

Adipose stem cells may also demonstrate increased retention when used in adipose transplantation for cosmetic reasons. Tanikawa et al. aimed to restore proper contour and volume to 18 patients with craniofacial microsomia using aspirated fat tissue supplemented with stromal adipose cells. The stromal adipose cells contain “multiple types of stem and regenerative cells” which is thought to compensate for the lower number of such cells present in the aspirated fat when compared to the integral undisturbed tissue [78]. Cellular and clinical analysis as well as radiographic assessment of preoperative and postoperative tissue volume and thickness was used to determine significance with the experimental group showing significantly better retention and volume than the control group. It has been suggested by Tanikawa et al. that the addition of stromal adipose cells contains multiple regenerative cell types allowing for better integration, improved quality, and increasing both angiogenesis and preadipocyte differentiation [78]. The study also found the cost of adding the stromal adipose cells to be entirely feasible. The only limitation to the study was the high dropout rate; however the remaining patients were found to have no complaints one year postoperatively.

Several studies have displayed significant success with improving graft retention via ASCs incorporation; however a review by Kokai et al. discusses several other studies which were unable to find any significant differences in appearance or patient satisfaction [76–79]. However it was mentioned that this may be due to differences in cell dose efficacy or in the treatment and refinement of the ASCs prior to implantation. The review also suggests the use of more animal studies with immune competent animals to determine the possible immune interactions between the recipient host and the lipoaspirate [79]. In summary, the use of adipose derived MSCs may be a wonderful supplement to conventional fat grafting but prospective research is needed to define its efficacy.

### 4.3. Muscle Regeneration and Possible Myogenic SCs

Muscular tissue has many essential visible functions in the craniofacial region. Its basic function is the production of contractile forces and eliciting movement. This basic function has a wide variety of uses: providing facial expression, mastication, speech, sight, respiration, swallowing, and many more. These activities are essential to a good quality of life; therefore regeneration of craniofacial muscle would make an outstanding impact in our treatment of Bell's palsy, neoplastic resection, craniofacial trauma, hemifacial atrophy, Moebius, and congenital deformities [1].

One common problem in many craniofacial abnormalities, particularly cleft lip/palate reconstruction, is recreating the normal functional capacity of the oral cavity (including swallowing, speech, and sucking) due to fibrosis and poor muscle presence leading to velopharyngeal dysfunction. Therefore muscle regeneration of the oral cavity, lips, and palate is imperative to ensure complete functionality and improve the patient's quality of life. Currently a suitable animal model using Sprague-Daley rats for regenerating palatal muscle following cleft palate has to be outlined but no current studies have yet been published [80]. Once studies begin to form they must overcome several key barriers such as the following:

1. Poor intrinsic regenerative capacity of palatal musculature poor muscle function of existing palatal musculature due to disorganisation and low myofibre count.
2. Fibrosis resulting from surgical intervention impairing and sort of muscle regeneration.

Stem cells seem like an ideal solution to overcoming these barriers due to their ability to immunomodulate, their stimulation of endogenous healing, and their promotion of fibroregulation.

Currently there is much research into the use of different types of SCs to regenerate muscle, each possessing differing characteristics (Table 3).

The discussion of which stem cell type is ideally situated for muscle regeneration similar to other tissues is a complicated and multifaceted one. The optimal choice of cell, scaffold, and delivery method has yet to be definitively discovered, as each method presents with various unique characteristics.

Clinical trials utilising a promising stem cell choice, mesoangioblasts, and the supposed most effective method of stem cell delivery have shown disappointing results [81, 82]. However recent developments are beginning to reveal light at the end of the tunnel. Through reprogramming mesoangioblasts to become iPSCs, Quattrocelli hoped that the cells would safely maintain their myogenic propensity while presenting all the obvious advantages of iPSCs. In order to determine whether the myogenic preference would affect the cells regenerative ability, cells were then differentiated into mesodermal lineages. Regenerative aptitude was examined using canine and murine cells in a dystrophic murine model as well as human cells in vitro. Analysis revealed that the cells showed the ability to regenerate skeletal striated muscle with engrafting of both slow and fast twitch muscle types. These cells also demonstrated successful correction of a muscular dystrophy murine model with in vivo regeneration of striatal muscle, contraction force, and function comparable to a control model. In vitro experimentation using human cells also exhibited a similar propensity for myogenesis [83].

Other methods of overcoming the dystrophic mouse model have also been discussed such as combined therapy utilising stem cell and gene therapy to achieve some exceptional results. Fibroblasts from dystrophic mice were reprogrammed to iPSCs which were then supplemented with microutrophin gene. Myogenic potential was induced by supplementation of Pax-3 and Pax-7. Following transplantation, utrophin positive myofibres and partial restoration of the dystrophin glycoprotein complex were observed and repopulation of the stem cell niche was recorded. Through the successful combination of gene and stem cell therapy systemic delivery of utrophin was confirmed resulting in markedly improved muscle function [84]. This study shows that many novel solutions exist for muscle regeneration and much further research is needed. Such models could be translated to the craniofacial region to provide a wide variety of potentially exciting future treatment options.

While the regeneration of striated muscle is critical for future craniofacial regenerative treatments, smooth muscle regeneration could present value towards vascular repair and reconstruction of the iris and ciliary muscles of the eyes. Contrary to the previous studies mentioned, Song et al. sought to regenerate smooth muscle utilising cells from the craniofacial region. DPSCs were induced to form bladder smooth muscle cells using a conditioned medium and TGF-*β*1. Effective differentiation was achieved with the transformed cells demonstrating morphological change indicative of smooth muscle cells along with the expression of smooth muscle markers such as *α*-SMA, desmin, myosin, and calponin [85]. While this study concentrated on DPSC use for bladder tissue engineering, they highlighted that the coculture method has the capacity to guide MSCs towards vascular smooth muscle cells and striated muscle cells. Both the neural crest cell origin of DPSCs and the previous successes seen by this coculture method suggest that this technique could possess clinical application in the craniofacial region.

In order for muscle regeneration to develop into a clinical treatment, numerous obstacles must be climbed. All current stem cell choices for regeneration are limited by certain complications such as poor expansion (satellite cells), safety (iPS cells), and a current lack of literature on the topic (dental stem cells). For dynamic tissues such as muscle tissue, scaffold design is paramount for acquiring the maximum regenerative potential from stem cell therapy. Currently required are new generations of scaffolds and biomaterials which intelligently amplify cell activity at a necessary location while engaging with the host response and native ECM to direct repair. Innovative materials combined with a greater understanding and improved manipulation of myogenic stem cells should provide future trials with a much greater likelihood of success [86].

### 4.4. Stem Cell Based Regeneration of Neuronal Tissues

The craniofacial nervous system is incredibly complex innervating multiple discrete tissues and carrying a vast array of intricate efferent sensory information to the CNS. Damage to any of the cranial nerves from congenital, idiopathic, or traumatic means can cause significant morbidities and loss of facial function. Structures which lose innervation have the tendency to rapidly atrophy; consequently prompt regenerative techniques could hold promise reducing tissue atrophy as well as improving function. The standard procedure now consists of autologous nerve grafts, which present the complications of nerve sacrifice somewhere else in the body [87].

Similarly to many tissues the exact ideal stem cell type for neural regeneration has not yet been discovered. Comparison of the neural regenerative capacity of MSCs, ASCs, and Schwann cells in fibrin nerve conduits in the sciatic nerve of Sprague-Dawley rats revealed that each enhances regeneration over the control as expected. However Schwann cells outperformed MSCs and ASCs, which was thought to be due to the release of neural bioactive factors. Unfortunately Schwann cells present numerous clinical difficulties such as culturing in vitro and the need for a biopsy/nerve sacrifice which limits their clinical applications until further cell manipulation is possible [87].

Many studies attempting neural regeneration have begun incorporating neurotrophic growth factors to enhance bioactive integration and regeneration. Hernández-Cortés et al. incorporated vasoactive intestinal peptide (VIP) as it has a wide range of functions such as vasodilation, immunomodulation, and neuroprotection and previous studies by Rayan et al. and Zhang et al. were able to demonstrate that VIP when delivered locally to a transected sciatic nerve was able to increase remyelination and increase the rate of axonal regeneration [88–90]. ASCs obtained from Balb/C mice were implanted with a lentiviral VIP vector forming ASC-VIP cells possessing typical MSC characteristics while also being capable of sustained VIP production greater than synthetic counterparts. The cell suspension was then implanted into D1-lactic-*ε*-caprolactone conduits which were subsequently implanted into the 10 mm sciatic nerve defect. Functional assessment of the ASC-VIP seeded conduit showed significantly improved recovery with substantially significant nerve regeneration and improved myelination. This increase in nerve regeneration is thought to explain the concurrent reduced muscle fibre loss in the experimental group [88]. Despite this study showing promising nerve regeneration, there were no coinciding effects on the targeted muscles and this was due to misdirection of regenerating nerve fibres and concurrent innervation of functionally incorrect muscles.

Other growth factors have also shown promise in aiding neural regeneration. Fibroblast-growth factor (FGF) has been shown to stimulate proliferation of glial cells and fibroblasts as well as stimulating angiogenesis [91, 92]. By morphing mouse iPSCs into primary and successive secondary neurospheres, Ikeda et al. aimed to develop and harvest glial progenitors. The secondary neurospheres were then seeded onto a nerve conduit formed of polylactic acid with poly *ε*-caprolactone incorporated interiorly. Human recombinant bFGF was then incorporated into gelatin microspheres to provide a slow release drug delivery system. C57BL6 mice had 5 mm artificial defects created in the sciatic nerve which were bridged using the experimental neurosphere/bFGF seeded conduits [91]. Motor recovery and analysis of neurofilament positive axons revealed autografting performed significantly better than the experimental group. However sensory testing and analysis of S-100 positive axons revealed comparable results [91]. While results comparable to autografting demonstrate another promising method of peripheral nerve regeneration, Ikeda et al. stated that the mechanisms of regeneration are unclear meaning that further elucidation of its methods is necessary to potentially allow for further manipulation and improvements as needed.

The use of dental stem cells was considered through the use of SHED cells seeded onto a “poly(e-caprolactone)/gelatin nanofibrous nerve guide” to repair a 10 mm artificial sciatic nerve defect in Wistar rats [93]. The scaffold used in this study was formed through electrospinning nanofibres of PCL/gel compound to create a high surface area to volume ratio allowing for improved cell adhesion. Analysis of functional motor and sensory recovery as well as axonal regeneration revealed greater improvement in the experimental group over any of the control groups. Histological analysis revealed increased presence of neural cell markers and a decreased expression of stem cell markers indicating that the SHED were entirely capable of transitioning into neural stem cells. The neural crest origin of dental stem cells is thought to provide them with additional in vivo neural propensity [93].

Dental pulp is known to contain both Schwann cells and stem cells and therefore may possess many ideal properties for nerve regeneration [94]. Sasaki et al. sought to explore the regenerative properties of dental pulp cells in a craniofacial setting. They designed a model using dental pulp cells (DPCs) suspended in a collagen gel which was infused into a 10 mm silicone tube. Nerve dissection was carried out producing a bilateral 7 mm gap in the buccal division of the facial nerve. The resected stumps were then sutured into the tube and after the designated time period the implanted tube was excised to allow for analysis [94, 95].

Tubulation of dental pulp cells was to confer functional myelinated axonal growth, improve recovery times, and produce measurable promotive levels of important neural growth factors such as NGF, BDNF, and GDNF. This comparably simple procedure showed that dental pulp cells have great potential in neural regeneration, with both DPSCs and dental pulp derived Schwann and endothelial cells detected [94]. In later experimentation Sasaki sought to exclusively utilise DPCs in the same model and carry out electrophysiological and functional analysis. Analysis of facial palsy at the end of the study revealed no significant differences between the DPC and autograft group. Investigation of action potentials revealed consistent electrophysiological results comparable to autografting. Tubulation shows some promise as an effective therapy as evidenced by the studies discussed; however conductive resorbable hybrid or biological polymer hybrids are necessary in order to facilitate a one-step surgical procedure [95].

A fascinating approach was utilised by Saito et al. where they opted to manipulate the stem cell through culture to form a 3D construct without the need for external materials. They performed thorough enzymatic extraction and purification of skeletal-muscle stem cells (SK-mSCs) while maintaining maximal cellular contacts. The resulting SK-mSC sheets were then centrifuged to form a 3D pellet. Saito et al. also opted to evaluate the pellets to regenerate a facial resection model, consisting of a “large facial nerve-blood vessel network deficit” encompassing both the buccal and zygomatic branches of the facial nerve [96]. The engrafted construct bridged the defect regenerating functioning myelinated axons along with supportive cell types. SK-mSCs were also found to have an angiogenic propensity generating smooth muscle cells and endothelial cells. Physiological nerve bridging without specific guidance was believed to be due to the spatial release of neurotrophic factors by resected stumps during early SK-mSC differentiation [96]. This regenerative model seems to hold huge promise in cases of craniofacial cancers. Wide tumour resections in the head and neck region are associated with marked decrease in quality of life; therefore any early promising studies targeting multitissue defects in the craniofacial area are vital and should be progressed upon.

Tissue regeneration must always occur in a controlled directional fashion; nowhere is this more uniquely evident than in neural regeneration. Future scaffold design and tissue engineering efforts need to concentrate on inductive strategies for improved directional proliferation over critical sized defects. Currently evidenced in the literature are several extremely promising stem cell sources with excellent neurogenic propensity. Dental stem cells are no exception to this group, displaying excellent neurogenic potential, low morbidity of harvest, and neuroprotective effects. DPSCs have shown significant aptitude, displaying the ability to ameliorate a wide variety of neural defect locations such as the optic nerve, spinal cord, and central nervous system [97–99]. Such widespread applications only act to further highlight their promise in this field.

## 5. Stem Cell Therapy for Sensory Regeneration

### 5.1. Early Steps in Taste Regeneration

Most loss in taste is due to neurological damage to the lingual and glossopharyngeal nerve; however certain conditions can also damage the taste buds leading to ageusia. Loss of taste and indeed any sense comes with certain loss of life quality. A study carried out by Takeda et al. discovered the taste bud progenitor cell which was identified by expression of the Lgr5 receptor; they then demonstrated that this type of cell could give rise to taste buds on the anterior and posterior sections of the tongue and demonstrated the cell's ability to regenerate posterior taste buds after CN-IX transection [100]. While this may be a promising discovery, this is the first reported regeneration of taste buds and more research is needed to quantify the value of this report.

### 5.2. Stem Cell Based Regeneration of Corneal and Retinal Tissues

Possibly the most damaging sense to lose is sight. The cornea is the most exposed part of the eye and it is most prone to injury from thermal or chemical trauma. If the limbus of the cornea is damage, there is significant epithelialisation which leads to stromal scarring and corneal opacity. A study by Rama et al. collected autologous limbal stem cells (LSCs) from the uninjured contralateral eyes of burn patients. Their results showed that LSCs can restore a transparent cornea with visual acuity in burn patients. However the efficacy of this treatment is dependent on % p63 bright cells as patients with received cultures with >3% p63 cells had a 78% success rate while those with cultures of <3% p63 cells had a success rate of less than 11% [101]. Therefore perhaps future studies should consider methods of optimising the culture and expansion conditions of limbal stem cells to isolate a suitable concentrate p63 expressing cells.

Another trial investigating corneal regeneration opted to utilise oral mucosal sheets in corneal reconstruction. Nishida et al. discuss how oral mucosa expresses keratin 3 similarly to the cornea; the excision of such tissue presents very low morbidity and the transplantation of autologous tissue avoids immunity complications in conditions such as Stevens-Johnson syndrome and ocular pemphigoid. Each of these qualities combined with an elevated native epithelial stem and progenitor cell population make it an attractive tissue choice. The oral mucosal tissue was cultured to transform into autologous epithelial cell sheets preimplantation. Transplanted oral epithelial cell sheets integrated into the corneal tissues well, becoming transparent and smooth. Visual acuity was described as improving “remarkably” and there were no postoperative complications. This trial seems to have presented a very suitable noninvasive, viable treatment for reconstructing the ocular surface [102].

Irreversible blindness may also be caused by photoreceptor loss in retinal disease. The retina proves to be difficult to provide regenerative care for as stem cell therapy had proved relatively ineffective. MacLaren et al. were able to demonstrate that an adult retina can integrate with rod photoreceptor progenitor cells expressing Nrl [103]. From this Pearson et al. developed a potential treatment strategy to improve vision via photoreceptor transplantation. Nrl-GFP+ rod precursors were extracted from postnatal mice which were then transplanted into a-transducin rod negative Gnat1 mice. Following integration of the rod precursors, analysis of vision revealed that the Nrl-GFP1 transplantation group was photosensitive with improved visual acuity and displayed visually guided behaviour. While in the early stages, this treatment holds huge potential in the future treatment of retinal disease [104].

Current research is beginning to recognise stem cell markers which identify promising stem cell and transit-amplifying populations for regeneration of ocular tissues. Current treatments for severe corneal damage involve transplantation which require donor tissue and suffer relatively high risks of rejections. Treatment options are similarly lacking for retinal diseases. There is an obvious apparent need for efficient conventional treatment for such ophthalmic conditions and early research shows that stem cell therapy has the potential to deliver. Interestingly Nishida et al. were able to demonstrate that dental stem cells may hold regenerative capabilities in ocular tissues. This relatively simple treatment model displayed high efficacy and with further development could present some really attractive results.

## 6. Regeneration of Exocrine Glands

In the craniofacial region exocrine glands such as the lacrimal or salivary glands carry out essential secretory functions. Loss or absence of function of these glands is associated with lower life quality and numerous difficulties. Exocrine glands are vulnerable to the damaging effects of radiotherapy but can also be damaged via trauma, disease, or congenital abnormality. Current treatments to restore the loss of secretion associated with gland hypofunction involve the use of artificial substitutes or stimulants (muscarinic receptor agonists) both of which may be costly and/or have undesirable side effects [105, 106].

### 6.1. Stem Cell Therapy to Improve Salivary Function

Salivary gland reconstruction may be needed due to trauma, radiotherapy, and tumour excision. Conditions such as xerostomia or Sjögrens syndromes are associated with considerable morbidity such as an increased caries incidence, difficulty in mastication, speech, and swallowing as well as burning mouth syndrome. Current treatments are noncurative. Nanduri et al. transplanted cells carrying the possible stem/progenitor markers, “c-kit, CD133, CD49f, and CD24,” into irradiated mice's salivary glands [105]. The mice transplanted with CD133 and c-kit displayed a significant increase in salivary output when compared to the control irradiated mice [105]. While this study showed promise in using a local stem cell population to improve salivary output, there exist some flaws. Radiotherapy can damage progenitor cell populations, preventing the harvesting of autogenous stem cell populations. This study could provide methods of providing treatment options for cases of salivary gland hypofunction caused by factors other than radiotherapy but may not possess much clinical feasibility.

A study that may possess greater efficacy was that carried out by Lim et al. which utilised the multipotency of ASCs. They set out to explore whether systemic delivery of xenogeneic ASCs can mitigate the deleterious effects of irradiated salivary glands in an animal model. ASCs were obtained from cultured surplus banked stem cells. ASCs were then injected into the tail vein of the control and the irradiated C3H female mice immediately after radiation. Salivary output in the ASC group was shown to have a statistically significant improvement on control irradiated group. This improvement in the ASC group was also seen at a molecular level with statistically significant rises in both mucin and amylase production over the control irradiated group. Analysis of the number of TUNEL-positive apoptotic cells at 4 weeks revealed that ASCs have a significant effect as to reducing tissue damage [107]. Analysis also revealed that the human ASCs were capable of transdifferentiating in salivary gland cells. However it was agreed by Lim et al. that the improvement in salivary gland function was most likely due to the paracrine signalling effects of bioactive factors released from the ASCs and not due to their direct transdifferentiation [106]. More conclusive animal models need to be developed and more efficient methods of cell delivery need to be established for more effective results to be obtained.

Early study models utilising stem cell therapy have shown promise and more recent studies attempting to generate a bioengineered salivary gland from isolated epithelial and mesenchymal stem cells display significant salivary flow and recovery of dry-mouth symptoms [108]. Our current research has not displayed any procedures using dental stem cells in salivary gland regeneration yet. This is surprising due to their ease of access and shared craniofacial origin; hopefully future studies will opt to include dental stem cells.

### 6.2. Lacrimal Gland Regeneration

The lacrimal gland is an essential component to complete vision. The lacrimal gland produces a serous secretion to maintain the tear film, manage the transparency of the cornea, and ensure that superior quality images are transmitted to the retina [95]. Similar to the salivary glands any disturbance to the lacrimal gland function termed “dry eye syndrome” can have some severe chronic effects causing discomfort and eventual loss of visual acuity [109]. Current treatments remain supportive and noncurative, consisting mostly of long-term frequent administration of lubricating eye drops. More advanced treatments such as autotransplantation of salivary glands are also showing inadequate success. Analogous to salivary glands, lacrimal glands are commonly damaged or disrupted via radiotherapy, trauma, systemic conditions, and so forth. Regenerative medicine may be able to provide a supportive treatment option to restore function [109].

Hirayama et al. proposed a more complete treatment consisting of a bioengineered lacrimal gland germ to restore function in lacrimal gland defect mice. The bioengineered lacrimal gland germ was fabricated through obtaining ED16.5 mice lacrimal gland germs and then separating the germ into respective epithelial and mesenchymal cell types. These cell types were then reconstituted into the appropriate germ morphology using a method of 3D cell manipulation. The extraorbital (as opposed to the intraorbital) lacrimal gland was excised in 7-week-old mice and the bioengineered lacrimal gland was inserted and connected to the original duct via a polyglycolic acid monofilament [110]. Histological analysis revealed correct 3D shaping with innervation following transplantation. Analysis of tear secretion after administration of pilocarpine revealed that the bioengineered mouse group showed no significant statistical difference in secretion rates to a control mouse. Analysis of tear components showed that the bioengineered lacrimal gland was entirely capable of producing appropriate tear proteins. Analysis of the corneal surface too showed that the bioengineered graft mice matched the control mice, proving that the bioengineered lacrimal gland model is capable of ensuring a healthy ocular surface. This study demonstrated an experimental model and treatment which could have potential as a surgical method for the treatment of dry eye disease. While this method provided correct physiological function, it is limited by its success rate of engraftment, its feasibility, and the invasiveness of the procedure [110]. While a long way from clinical trials, current studies into lacrimal gland and organ regeneration present hopeful results and stem cell therapy could provide many future treatment modalities.

## 7. Frontier of Stem Cell Therapy, What Does the Future Hold?

The future of stem cell therapy in the craniofacial medicine is bright and optimistic with huge opportunities for improvement and a vast array of upcoming possibilities. In future years to come, further research will reveal new approaches towards regeneration while expanding our knowledge of the current field, hopefully allowing for exceptional clinical results. Our developing comprehension of signalling pathways, cell manipulation, and stem cell characteristics may allow us to circumvent current treatment morbidities and discover methods of ameliorating conditions considered incurable. Most current studies on animal models evaluate a single promotive stem cell type in healing. Future studies may be able to proceed with multiple cell types and manipulate a wide variety of introduced and native factors over extended periods of time to create the ideal signalling network for optimum regeneration. However before breakthroughs like this are made it is essential that the action of these molecules inside signalling pathways be fully comprehended to ensure that there are no potential negative side effects. Future research into growth factors must also overlap with tissue engineering and evolving scaffold design so that drug kinetics can be more efficiently controlled and monitored. Despite the current shortcomings, it seems without doubt that small promotive factors will partake in the future of regenerative medicine [111].

Stem cell exploration is revealing an exciting tomorrow where grievous traumatic injuries can be treated safely with no loss of function or aesthetics. Huge breakthroughs have been made in the field in the past decade particularly with the generation of iPSCs from adult human somatic cells types [112, 113]. The reprogramming of adult human cells into various different stem and progenitor cell types has huge implications in the field of stem cell therapy as it solves many long-term problems related to other stem cell types. As iPSCs are patient specific, they are not burdened by immunological defences unlike stem cells from allogenic or xenogenic sources. iPSCs are also benefited by their potential abundance and versatility as they can be harvested easily and cheaply to develop into a wide variety of different tissue cell types [112, 113]. This ability to differentiate to several different tissue types is ideal for craniofacial reconstruction as there is a wide variety of tissue types in need of regeneration following such a procedure. Research into iPSCs is still only scratching the surface; future experimentation and research should reveal enhanced results and their potential will no doubt continue to be exploited and expanded [113].

The generation of iPSCs was only possible through the mechanisms of gene therapy, an equally promising tool in the field of craniofacial regeneration. Aside from reprogramming somatic cells into stem cells, gene therapy has numerous other possibilities in the field of regenerative medicine.

1. Head and neck cancers: research into gene therapy has resulted in the formation of oncolytic viruses which specifically target replicating tumour cells to induce cell death and a corresponding decline in tumour size. While this form of treatment may not be directly involved with the reconstructive process, it could have a definite impact on the scale of reconstruction/regeneration needed [114].
2. Mineralised tissue regeneration: gene therapy research has revealed some fruition in mineralised tissue regeneration through controlled spatial activation of osteogenic genes and the resulting upregulated proteins. This method of growth factor and protein delivery can greatly increase regeneration especially when combined with other regenerative techniques such as the introduction of stem cells [4, 114].
3. Salivary gland function: following ablative radiotherapy, certain medications, and so forth, salivary glands often cease to function properly. Using gene therapy, aquaporins can be introduced into the ductal epithelial cells allowing for increased fluid movement. Human clinical trials using this therapy have been found to be promising, with subjective improvements in xerostomia and relieved symptoms [114, 115].

Although large-scale craniofacial reconstruction is not currently benefitted by gene therapy, its applications of controlled protein production and tissue repair coordination make it an invaluable tool in the advancement of regeneration and will present as an adjunctive therapy to stem cell based treatments [114].

Even the development and advancement of available technologies are allowing us to produce scaffolds and other tissue engineering materials with superb biocompatibility and mechanical properties. Future biomaterials and scaffold designs are assured to increasingly mimic the extracellular matrix and further engage and enhance stem cell activity providing yet another boost to this field.

This is a brief view of some of the most promising methods, techniques, or materials set to improve and expand the field of stem cell therapy. The future for stem cell therapy is not only bright in terms of upcoming treatment options for clinicians. New laws and regulations regarding stem cells and gene therapy approaches are allowing for new avenues of research. This sort of government backing along with financial interest from the private sector will ensure continued prosperity in the research of new treatments. Research is even being carried out on the feasibility of current regenerative techniques showing huge private marker backing [116]. Research into regenerative medicine is now also reaching the phases of clinical trials in many cases which will hopefully result in the introduction of new effective regenerative treatments in the near future.

## 8. Conclusion

Throughout this paper, there have been many demonstrations of the ground-breaking research, studies, and trials into future and potential stem cell treatments applications for a wide range of conditions, injuries, and defects. Areas of stem cell research show possible promising treatments in regard to nearly every craniofacial structure.  Table 4 presents a summary of the especially promising stem cell types presented in this paper.

Dental stem cells are an upcoming source of stem cells in regenerative medicine. Current evidence shows a broad range of promising evidence in regenerating craniofacial tissues. Their use has shown significant improvement in function and healing rate and they are associated with much lower rates of harvest morbidity and postoperative discomfort. Ultimately future research will determine definitive uses of dental stem cells within the field of stem cell therapy; however it seems that their future is optimistic.

## Figures and Tables

**Table 1:** Methods of stem cell classification.

Stem cell classification				
Method of classification	Source	Origin	Differentiation potential	References
1	Autogeneic	Embryonic	Totipotent	[6]
2	Allogeneic	Foetal	Pluripotent	
3	Xenogeneic	Perinatal	Multipotent	
4		Adult	Oligopotent	
5		Induced	Unipotent	

**Table 2:** The different types of dental stem cells and their potential applications in regenerative medicine are displayed.

Dental stem cells	Potential applications	References
DPSCs	Dentine-pulp regeneration, PDL regeneration, and nonoral tissue regeneration, for example, bladder tissue engineering	[4, 10, 85]
SHEDs	Dentine-pulp regeneration, craniofacial bone regeneration, neural tissue regeneration, and nonoral tissue regeneration, for example, hepatocyte-like cells	[10, 35, 93]
SCAPs	Dentine/bone regeneration, continued root formation, and bioroot engineering	[10]
PDLSCs	Periodontal regeneration	[10, 62–64]
DFPCs	Tooth root regeneration	[10]
GMSCs	Wound healing and immunomodulatory therapies for inflammatory disease	[10]

**Table 3:** This table shows potential SCs for use in muscle regeneration and their positive and negative characteristics.

Stem cell	Advantages	Disadvantages	References
Embryonic stem cells (ESCs)	(i) Have been shown to improve muscle function in vivo (ii) Pluripotent	(i) Research limited by regulations (ii) Need presence of box genes (Pax-7)	
Induced pluripotent stem cells (IPSCs)	(i) Less regulations (ii) Pluripotent	(i) More study needed to ensure that there is no tumorigenic potential	
Satellite cells	(i) Express Pax-7 (ii) Self-proliferating	(i) Difficult to isolate (ii) Damaged by in vitro culture	
Muscle derived stem cells (MDSCs)	(i) Osteogenic and adipogenic		[81, 117]
MSCs	(i) High ability to differentiate (ii) Modulate inflammation	(i) Need more research before in vivo studies	
Muscle derived CD133+ stem cells	(i) Formed myosin heavy chain (characteristic of craniofacial muscle) (ii) Safe and feasible	(i) Myogenesis in vitro requires additional cell cultures	
Mesoangioblasts	(i) Angiogenic (ii) Easy expansion in vitro	(i) Require factors to improve migration	

**Table 4:** This table presents a general summary of target tissues or organs in need of superior regenerative solutions and the corresponding stem cell types evidenced with feasible regenerative potential.

Tissue/tissues to be regenerated	Promising stem cell sources to be used in isolation or conjunction	References
Bone	BM-MSCs, ASCs, DPSCs, SHED	[24, 26, 34, 36, 37, 40, 42, 44, 46, 49, 50, 54, 56, 62–65, 71–73, 78, 81, 83–85, 91, 93, 94, 96, 101–104, 106, 117]
Cartilage	PDLSCs, BM-MSCs, TMJ-SCs	
Tooth	PDLSCs, BM-MSCs, iPSCs, and tooth progenitor cells	
Oral mucosa	Oral mucosa stem cells and iPSCs	
Periodontium	PDLSCs and iPSCs	
Skin	Embryonic MSCs, LGR 6+ SCs, PBSCs, and ASCs	
Fat	ASCs	
Muscle	Mesoangioblasts, iPSCs, DPSCs, and muscle stem cells	
Nerve	iPSCs, SHED, DPSCs, SK-mScs	
Cornea	LSCs, and oral mucosa epithelial stem cells	
Retina	NRl rod photoreceptor progenitor cells	
Salivary gland	CD133/c-kit expressing SCs and ASCs	
Lacrimal gland	ASCs	
